# Synaptic AP2 CCV life cycle regulation by the Eps15, ITSN1, Sgip1/AP2, synaptojanin1 interactome

**DOI:** 10.1038/s41598-021-87591-3

**Published:** 2021-04-13

**Authors:** R. Mishra, G. F. Sengül, E. Candiello, P. Schu

**Affiliations:** 1 Department of Cellular Biochemistry, University Medical Center, Georg-August University Göttingen, Humboldtallee 23, 37073 Göttingen, Germany; 2 Institute for Cancer Research and Treatment (IRCC), Turin, Italy; 3 Present Address: Department of Clinical Neurosciences, John Van Geest Centre for Brain Repair, University of Cambridge, Cambridge, England UK

**Keywords:** Biochemistry, Cell biology, Neuroscience

## Abstract

The AP1/σ1B knockout causes impaired synaptic vesicle recycling and enhanced protein sorting into endosomes, leading to severe intellectual disability. These disturbances in synaptic protein sorting induce as a secondary phenotype the upregulation of AP2 CCV mediated endocytosis. Synapses contain canonical AP2 CCV and AP2 CCV with a more stable coat and thus extended life time. In AP1/σ1B knockout synapses, pool sizes of both CCV classes are doubled. Additionally, stable CCV of the knockout are more stabilised than stable wt CCV. One mechanism responsible for enhanced CCV stabilisation is the reduction of synaptojanin1 CCV levels, the PI-4,5-P_2_ phosphatase essential for AP2 membrane dissociation. To identify mechanisms regulating synaptojanin1 recruitment, we compared synaptojanin1 CCV protein interactome levels and CCV protein interactions between both CCV classes from wt and knockout mice. We show that ITSN1 determines synaptojanin1 CCV levels. Sgip1/AP2 excess hinders synaptojanin1 binding to ITSN1, further lowering its levels. ITSN1 levels are determined by Eps15, not Eps15L1. In addition, the data reveal that reduced amounts of pacsin1 can be counter balanced by its enhanced activation. These data exemplify the complexity of CCV life cycle regulation and indicate how cargo proteins determine the life cycle of their CCV.

## Introduction

**Figure 1:**
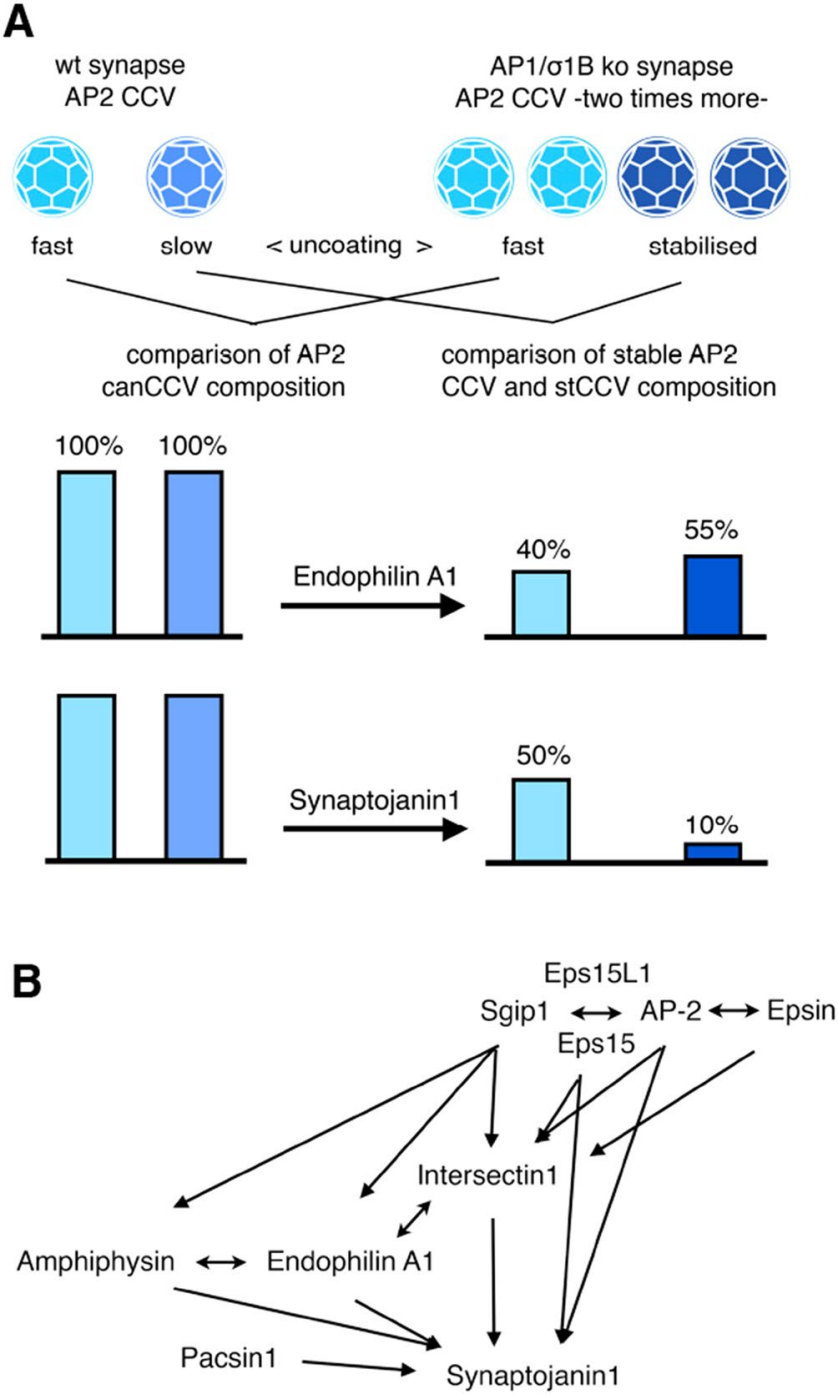
(**A**) Summary of alterations in the synaptic AP2 CCV pools, induced by impaired SV recycling due to AP1/σ1B-adaptin deficiency. Pool sizes of canonical canCCV and stable CCV are increased twofold and in addition, stable, slow uncoating CCV become more stabilised (stCCV) Bar diagrams indicate the protein reduction per CCV for endophilin and synaptojanin1. (**B**) The synaptojanin1 interactome of CCV proteins and their interactions. Clathrin and dynamin are not included. Proteins listed at the top appear first at a AP2 CCP. Both Eps15 proteins bind synaptojanin1. Pacsin1 belongs to the group of proteins recruited late during CCV budding.

Synapses contain two classes of AP2 CCV (adaptor-protein complex 2; clathrin-coated-vesicles). Canonical AP2 CCV, canCCV, and AP2 CCV with a stabilised protein coat and thus a longer half live. Stable CCV account for about 15% of synaptic AP2 CCV^1,2^. In synapses of the AP1/σ1B-adaptin knockout mouse (ko) numbers of both classes increase twofold. In addition, the stable AP2 CCV in ko mouse synapses are even more stabilised CCV, hereafter stCCV^1^, compared to wt stable CCV, as summarised in Fig. 1. AP2 stCCV do not endocytose synaptic vesicle (SV) proteins. They are enriched in the synaptic active zone (AZ) proteins stonin2 and Git1^1^. These are organised in circles around AZ. Stonin2, an AP2 co-adaptor CCV protein, recruits Git1^3–6^. Deficiency in presynaptic Git1 leads to impaired SV recycling and transsynaptic signaling^3^. The molecular mechanisms are not known. Git1 transport via stCCV points to important functions of stCCV in the regulation of AZ plasticity^1^. These changes in AP2 CCV pathways are a secondary phenotype of the AP1/σ1B ko. The AP1/σ1B ko causes slowed down and incomplete SV recycling and enhanced endolysosomal protein sorting^1,2,7,8^. Mice and humans deficient in the X-chromosome encoded AP1 subunit σ1B have severe mental retardation disease and deficits in motor control^8^. The alterations in the AP2 CCV pathways are expected to suppress the effects of disturbed synaptic protein sorting caused by AP1/σ1B-deficiency.

The AP1/σ1B ko induced secondary phenotype in AP2 CCV pathways enables us to investigate physiological mechanisms regulating the AP2 CCV life cycle. CCV coat protein ko and protein domain overexpression studies revealed coat protein interactions and functions in CCV formation. However, CCV proteins interact with several proteins during the CCV life cycle via the same domains and thus these approaches analyse early steps in CCV formation, but they can not resemble physiological mechanisms regulating the CCV life cycle.

We identified three molecular mechanisms responsible for the enhanced stability of AP2 stCCV^1^. They affect all layers of a CCV: the outer clathrin basket, the inner AP2 layer and the membrane phospholipid composition. Compared to wt stable CCV, stCCV have less of the clathrin disassembly protein Hsc70, their AP2 complexes are hyperactivated for stable membrane binding and they have less of the PI-4,5-P_2_ phosphatase synaptojanin1. AP2 hyperactivation is caused by an increase in AAK1 (adaptin-associated kinase 1), which phosphorylates μ2. This stabilises the AP2 open state, enabling μ2 to bind PI-4,5-P_2_ and also cargo^9–12^. A stCCV contains with 10% of wt only residual amounts of synaptojanin1. PI-4,5-P_2_ dephosphorylation by synaptojanin1 is essential for AP2 membrane dissociation^13–19^. Synaptojanin1 catalytic activity is stimulated by high membrane curvature^20^. Therefore, its CCV uncoating activity is regulated by its recruitment into CCV. In this study, we present our analysis of the mechanisms regulating synaptojanin1 levels in AP2 CCV.

Understanding the mechanisms regulating synaptojanin1 activities is not only important for CCV life cycle regulation, but is also essential to understand the development of several severe neurological diseases. Synaptojanin1 deficiencies cause neonatal refractory epilepsy and neurodegeneration^21^. Point mutations have been identified in patients with early onset Parkinson’s disease, intractable epilepsy and tau pathology^22–26^. Three forms of Alzheimer’s disease are associated with elevated synaptojanin1 levels^27–29^. Loss of its 5- and 4-phosphatase activities leads to early onset refractory seizures and neurological decline^21^. Its overexpression is associated with Down syndrome^30,31^. Its SAC1 domain dephosphorylates PI-4-P, but is also able to dephosphorylate PI-3-P and PI-3,4-P_2_, both of which regulate endosomal dynamics and autophagosome formation. Synaptojanin1 deficiencies induce strong phenotypes in SV recycling, whereas autophagosomes are not abundant in mature synapses^32,33^.

The synaptojanin1 CCV interactome consists of 9 proteins. Eight of these have interactions with each other, as summarised in Fig. 1. Synaptojanin1 can be bound by proteins appearing early at the site of CCV formation and also by proteins appearing late, just before membrane scission. Early proteins are AP2, Eps15, Eps15L1 (Eps15-like 1, also known as Eps15R) and clathrin^34–36^. Proteins appearing later are endophilin A1, amphiphysin, intersectin1 (ITSN1) and pacsin1 (also known as syndapin1). They also bind each other and those early appearing proteins. These proteins are also bound by the early appearing proteins Sgip1 and epsin (Fig. 1)^13,37–43^. It is not understood, why synaptojanin1 is bound by so many, ubiquitously expressed CCV proteins. This could ensure robust, continuous recruitment of synaptojanin1. The late appearing endophilin A1 (hereafter just endophilin) recruits synaptojanin1 just before membrane scission and thus this interaction has been considered to be the most relevant^13,41,42^. The late recruitment of an uncoating enzyme, whose catalytic activity is activated by high membrane curvature, also appears to be beneficial for CCV formation^13,44–46^.

Of all the synaptojanin1 interacting CCV proteins, we already analysed amphiphysin and endophilin levels in wt and ko synaptic AP2 CCV in a previous study^1^. A stCCV contains 50% of endophilin compared to a stable CCV from wt mice, but only 10% of synaptojanin1^1^. This indicates that endophilin does not determine the amount of synaptojanin1 recruited into an AP2 CCV. However, phosphorylation of endophilin by LRRK2 kinase could reduce its binding of synaptojanin1 causing the dramatic reduction in synaptojanin1 stCCV levels^40,47–51^. Here we show that the levels and activities of the endophilin modifying kinase LRRK2 are not changed in stCCV compared to wt stable CCV, finally excluding endophilin as the synaptojanin1 recruiter. To unravel the mechanisms regulating synaptojanin1 CCV levels, we analysed ko canCCV and stCCV for alterations in the remaining synaptojanin1 interactome members. ITSN1 CCV level determine synaptojanin1 CCV levels. Sgip1/AP2 competition with synaptojanin1 for ITSN1 binding can lower synaptojanin1 levels even further. ITSN1 CCV levels are determined by Eps15 CCV levels, but the highly homologous Eps15L1 is not involved. Thus, protein:protein ratios and competitive interactions determine the CCV life cycle. In addition, this study shows that a reduction in pacsin1 can be compensated by its enhanced activation. This demonstrates the existence of complex pathways regulating the CCV life cycle. A model is proposed, which shows how co-adaptors and thus cargo proteins determine the life cycle of their AP2 CCV.

## Results

### Endophilin and LRRK2 do not determine synaptojanin1 CCV levels

**Figure 2:**
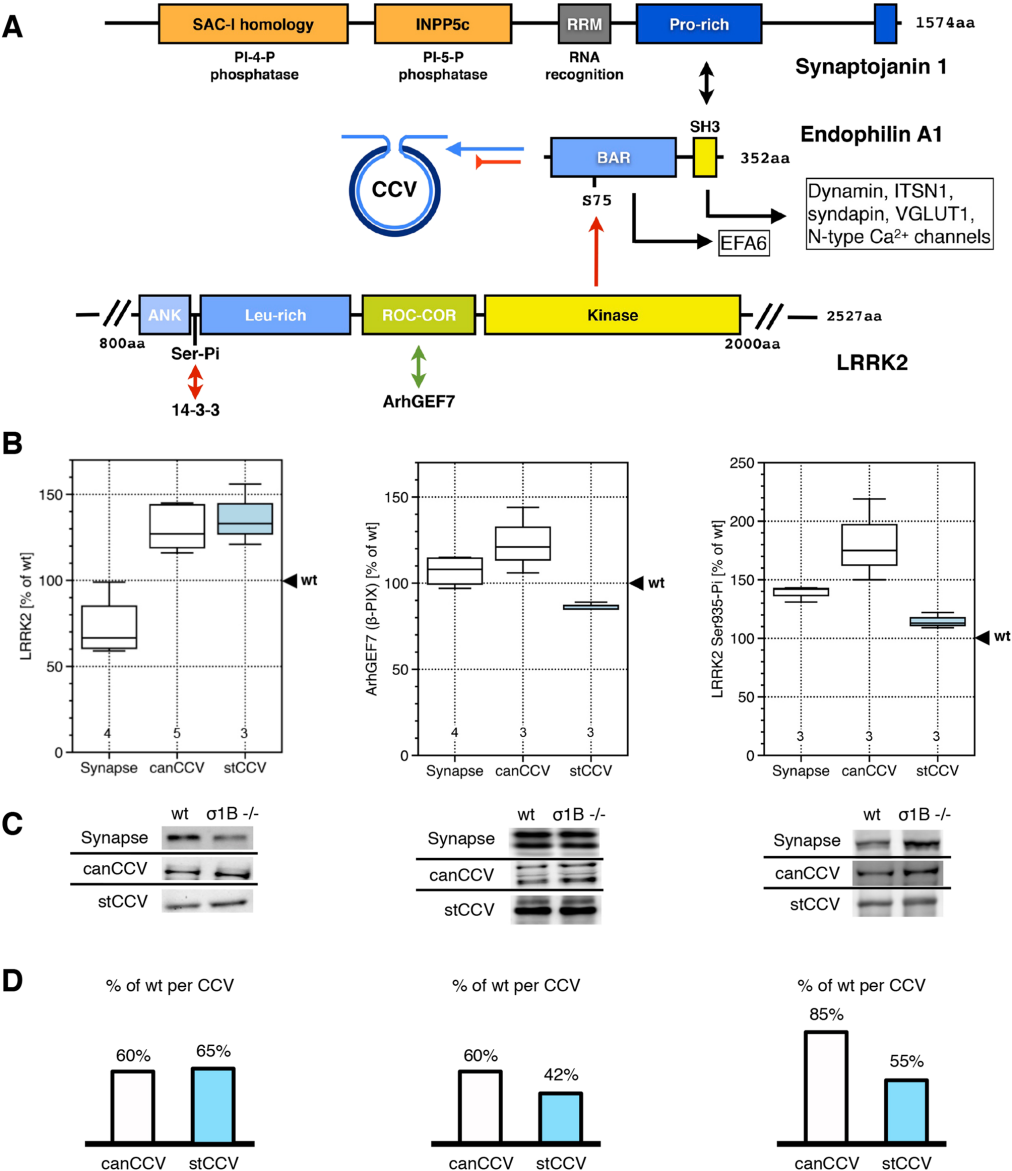
(**A**) CCV protein interactions of endophilin A1. Endophilin membrane binding to the neck of budding CCV, and synaptojanin1 recruitment, could be inhibited by LRRK2 (N-terminal armadillo and C-terminal WD40 domains are not depicted). If endophilin membrane binding is inhibited, it could also be recruited by binding of its SH3 domain to CCV proteins. Green arrow indicates activating, red arrows inhibitory reactions. (**B**) Comparison of LRRK2 protein (240 kDa band) and activity (ArhGEF7, 74 kDa band; LRRK2 Ser935-Pi, 240 kDa band) levels between synapses and synaptic AP2 canCCV and wt, stable CCV and stCCV pools from wt and AP1/σ1B-adaptin ko mice. Values from wt samples are defined as 100% and values from ko samples are expressed relative to wt. Numbers in box-blot diagrams indicate the number of independent biological samples. (**C**) Representative western-blot data used for the box-blot diagram (see “Materials and methods” section for details). (**D**) Bar scheme below indicates changes in protein levels per CCV.

We compare the protein content between the synaptic wt and ko canCCV as well as wt stable CCV and the ko stCCV. The numbers of both AP2 CCV classes are doubled in ko synapses^1,2,8^. Thus, a change in the amount of a protein in a single CCV is half of the change in the CCV pool (Fig. 1). Previously, we have shown that the synaptojanin1 binder endophilin is reduced down to 40% and 50% of wt levels in a canCCV and a stCCV of AP1/σ1B ko mice. Synaptojanin1 is also reduced to 50% of wt in canCCV, but a stCCV has only 10% of wt synaptojanin1 levels, despite 50% of wt endophilin levels^1^. This suggests that endophilin may not regulate the amount of synaptojanin1 incorporated into CCV. However, data in the literature indicate, that endophilin-synaptojanin1 binding might be differentially regulated by LRRK2 (leucine-rich repeat kinase)^41,42,52–54^. Endophilins BAR-domain binds the neck of a budding CCV, before membrane scission^55^, and its SH3-domain binds synaptojanin1 (Fig. 2A). The BAR-domain residue Ser75 is phosphorylated by LRRK2, but the function of this modification has not been tested directly^53^. It is expected, that Ser75 phosphorylation blocks membrane binding of the BAR-domain, but this would not necessarily inhibit endophilin recruitment to CCV, because its SH3-domain binds 6 CCV proteins: dynamin, intersectin1 (ITSN1), syndapin, VGLUT1, N-type Ca^2+^-channels and parkin^56–60^. If endophilin is recruited via its SH3-domain by CCV proteins, its BAR-domain can recruit the Arf6-GEF EFA6^61^ (Fig. 2A). The order of these binding modes is not known. A competition of 7 proteins for endophilins SH3-domain could impair its ability to recruit synaptojanin1. LRRK2 activity could be increased in stCCV favouring its recruitment via protein-SH3 binding and leading to reduced synaptojanin1 recruitment. Therefore, we compared LRRK2 levels and also its activation levels between the CCV from wt and ko mice.

AP1/σ1B ko synapses contain 65% of wt LRRK2 levels and their two CCV classes have ~ 70% of wt levels (Fig. 2), but LRRK2 activity could be stimulated in stCCV compared to wt stable CCV. LRRK2 has a GTPase domain and GTP binding activates kinase activity^52^. Importantly, the LRRK2 GEF ArhGEF7 binds Git1, which is enriched in stCCV, suggesting higher LRRK2 activity in stCCV^1,3^. The ArhGEF7 level is slightly increased in AP1/σ1B ko synapses and thus its synaptic levels do not limit LRRK2 activation. A canCCV of AP1/σ1B ko synapses has 60% of wt ArhGEF7 levels. A stCCV has ~ 42% of wt, indicating that stCCV LRRK2 is less active than canCCV LRRK2 (Fig. 2). The commercial anti-ArhGEF7 antibody labelled two protein bands in all fractions. The intensity of both changed in the same way. CCV were enriched in the fast migrating ArhGEF7 protein (Fig. 2C). LRRK2 phosphorylation, e.g. by PKA, on Ser935 leads to 14-3-3 protein binding and its inactivation (Fig. 2A)^62,63^. An AP1/σ1B ko synapse canCCV has 85% of wt LRRK2 Ser935-Pi-level. A stCCV has 55% of wt LRRK2 Ser935Pi, but LRRK2 is also less activated by ArhGEF7. Thus, LRRK2 activation/inactivation cycles appear to be less active in stCCV compared to wt, stable CCV. The endophilin CCV levels and the LRRK2 data demonstrate that endophilin does not determine the amount of synaptojanin1 in a CCV.

It is worth mentioning, that the changes in ko synapse LRRK2 levels are in line with their increase in endosomal protein sorting. Synapses of AP1/σ1B ko mice have 65% of wt LRRK2 levels and the LRRK2 Ser935Pi-level is increased to 140% of wt (Fig. 2). Decreased LRRK2 kinase activity is in line with the upregulation of endosomal protein transport, because LRRK2 inhibits several Rab proteins which regulate endosomal protein transport^52^.

### Pacsin1: reduced recruitment into stCCV, but enhanced activation

**Figure 3:**
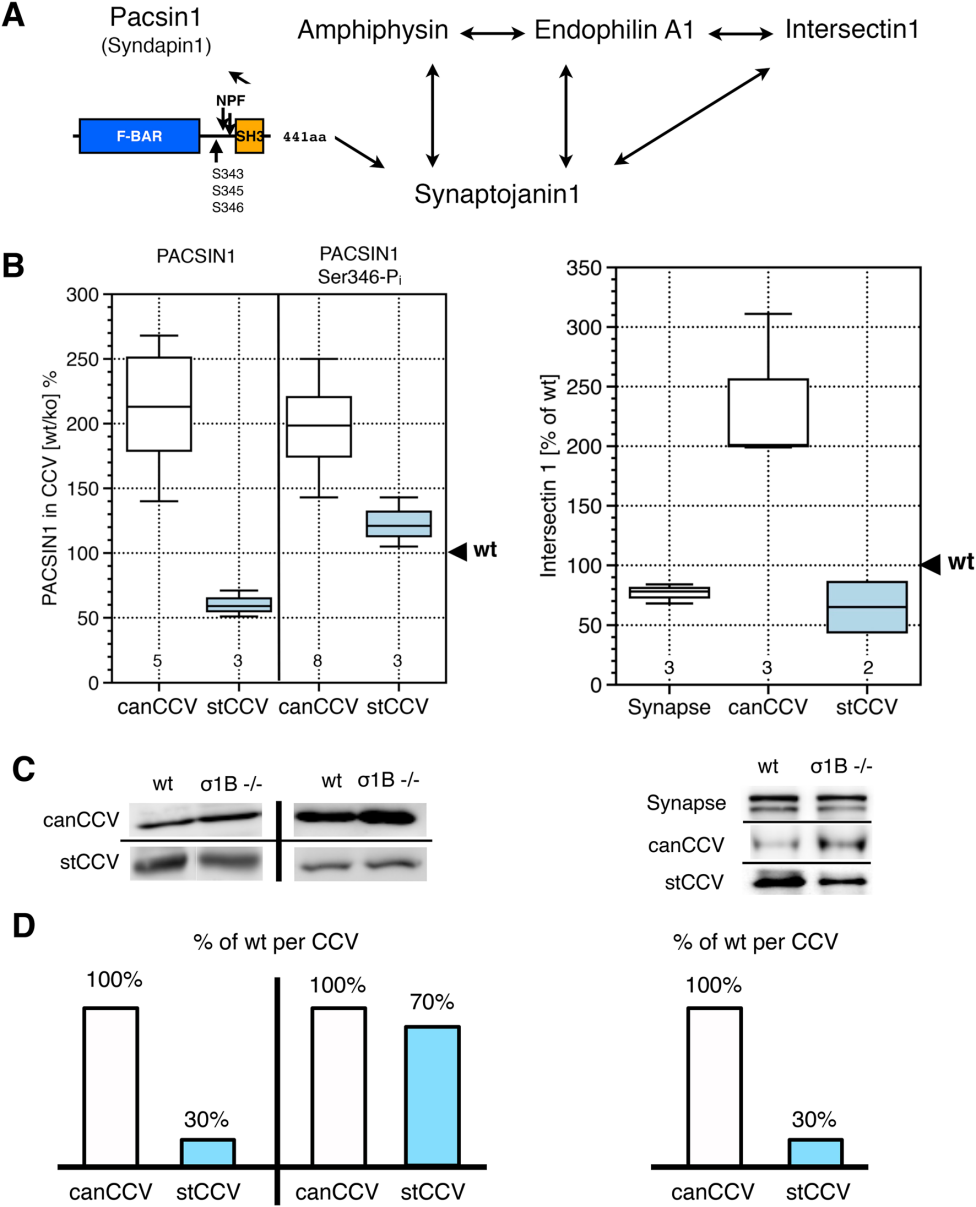
Pacsin1, activated pacsin1 (Ser346-Pi) and ITSN1 wt versus ko synaptic and CCV levels. (**A**) Protein–Protein interaction scheme of these synaptojanin1 binders. (**B**) Comparison of pacsin1, phosphorylated and activated pacsin1 and ITSN1 levels between synaptic AP2 CCV from wt and AP1/σ1B ko mice. Values from wt samples are defined as 100% and values from ko samples are expressed relative to wt. Numbers in box-blot diagrams indicate the number of independent biological samples. (**C**) Representative western-blot data used for the box-blot diagram (see “Materials and methods” section for details) Pacsin1, 52 kDa band; Pacsin1 Ser346-Pi, 52 kDa band; ITNS1, 200 kDa band;. (**D**) Bar scheme below indicates changes in protein levels per CCV.

The synaptojanin1 binder pacsin1 (Fig. 1) has a F-BAR, a SH3 and an unstructured domain (Fig. 3A). Its SH3-domain interacts with dynamin and the actin polymerisation inducer N-WASP. Intramolecular interaction of F-BAR and SH3 domains causes pacsin1 autoinhibition. The intermediate, unstructured sequence contains NPF-motifs, which mediate binding to EHD’s (epsin-homology-domain) of CCV co-adaptor proteins^64^. Pacsin1 autoinhibition is prevented by serine phosphorylation in this unstructured sequence. Ser343 is phosphorylated by Pak5, which also phosphorylates the proline-rich domain of synaptojanin1, with which it binds endophilins SH3 domain. Either of the two phosphorylations stabilises pacsin1-synaptojanin1 binding^39,50,65–70^.

AP1/σ1B ko synapses and their canCCV contain wt pacsin1 levels (Fig. 3). The commercial anti-pacsin1 antibody detected besides the major protein band a second band of very low intensity. Its changes in intensities between wt and ko samples matched those of the major band (Fig. 3C). A stCCV contains only 30% of wt, stable CCV pacsin1. This reduction could be responsible for the reduction in synaptojanin1 levels, but stCCV pacsin1 could also have a higher phosphorylation level.

Using a commercial pacsin1 Ser346-Pi antibody, we found wt pacsin1-Pi levels in ko mice canCCV (Fig. 3). A stCCV has 65% of wt pacsin1-Pi, despite the pacsin1 reduction down to 30% of wt (Fig. 3). Thus, the fraction of activated pacsin1 in a stCCV is about twofold higher than in a wt, stable CCV. We can not exclude, that the reduction in the stCCV pacsin1 level does not contribute at all to the reduced amount of synaptojanin1 in stCCV. However, the compensation of reduced pacsin1 stCCV levels by its increased activation is not in line with a pacsin1 function in synaptojanin1 recruitment. These data demonstrate a quality control mechanism for the CCV life cycle regulation. The kinase remains to be determined.

### ITSN1 is differentially recruited to CCV classes

**Figure 4:**
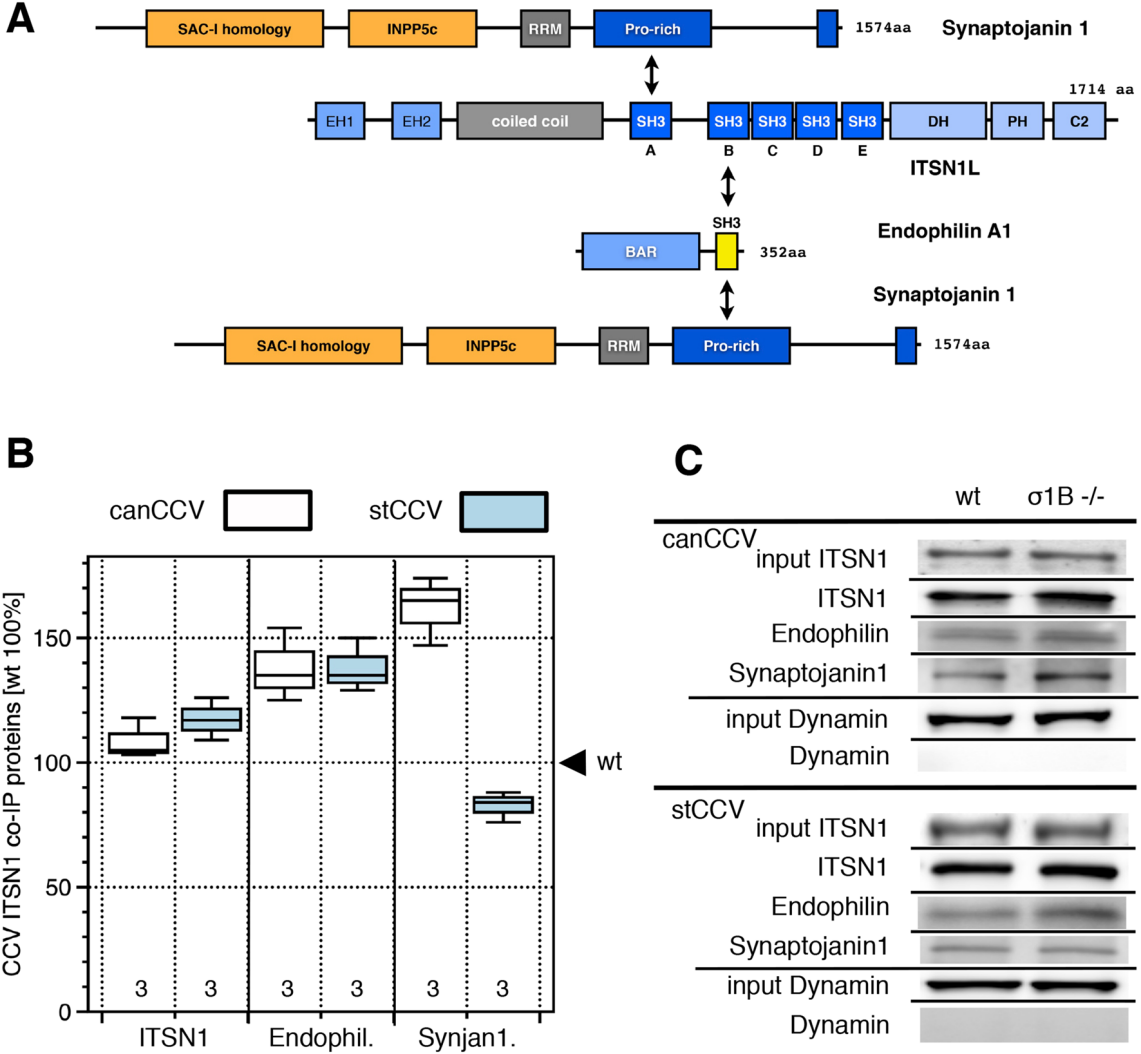
(**A**) Interaction of synaptojanin1 with ITSN1 and endophilin and of endophilin with ITSN1. (**B**) ITSN1 binding ratios of endophilin and synaptojanin1 of wt and ko canCCV and wt stable CCV and ko stCCV measured by ITSN1 immunoprecipitation after CCV coat protein solubilisation. Numbers indicate the number of independent experiments performed. (**C**) Representative western-blot experiments (see “Materials and methods” section for details). ITSN1, 200 kDa band; Endophilin, 37 kDa band; Synaptojanin1, 145 kDa band; Dynamin, 95 kDa band.

ITSN1 is able to bind synaptojanin1 and endophilin simultaneously and independent of each other. It has been proposed, that ITSN1 is a platform for coordinating synaptojanin1 recruitment by endophilin (Fig. 3A)^58^. ITSN1 appears early at budding CCV and its level increases continuously during budding. It could mediate an even synaptojanin1 distribution, supporting fast PI-4,5-P_2_ dephosphorylation. Brain ITSN1, ITSN1L, has a C-terminal extension of RhoGEF, PH and C2 domains. They are preceded by five SH3 domains (Fig. 4A). ITSN1L (hereafter just ITSN1) is constitutively active due to a five amino acid insertion in the SH3A domain^71,72^. ITSN1 SH3A binds synaptojanin1, SH3B binds endophilin. The linking sequence binds AP2 (Fig. 4A)^73,74^. AP1/σ1B ko mice synapses contain 80% of wt ITSN1 levels. The commercial ITSN1 antibody detects also a low intensity, faster migrating protein in synapses. It is also present at 80% of wt levels (Fig. 3C). A canCCV of AP1/σ1B ko mice has wt levels of ITSN1 (Fig. 3). A stCCV has only 30% of a wt, stable CCV ITSN1 (Fig. 3). The constitutively active ITSN1 is the only synaptojanin1 binding CCV protein, whose level is dramatically reduced in stCCV and thus ITSN1 appears to regulate synaptojanin1 CCV levels.

### Preferential synaptojanin1 binding by ITSN1

In order to verify preferential ITSN1-synaptojanin1 binding within a CCV, we solubilised the CCV coats, immunoprecipitated ITSN1 and compared the amounts of co-isolated endophilin and synaptojanin1 between wt and ko synapse CCV. CCV have to be purified at pH 6.4 to stabilise the coat*.* Shifting the pH to 7–8 induces disassembly and solubilisation of the proteins. Vesicles are removed by centrifugation. The pH of the protein solution was adjusted back to 6.4 to restore the condition, which stabilises coat protein interactions. The ITSN1 antibody binds the SH3A-SH3B linker domain, competing with AP2 for ITSN1 binding^73^. Isolated anti-ITSN1antibody-beads were washed three times at pH 6.4. Proteins were eluted by adding threefold concentrated, dye-containing, non-reducing SDS-PAGE loading buffer. The amounts of proteins eluted from ko CCV ITSN1-beads were compared to those from wt CCV, also here defined as 100%. Dynamin was not isolated (Fig. 4), indicating that ITSN1 and the co-isolated proteins are not part of a large protein network.

The wt and ko mouse canCCV have the same amount of ITSN1 (Fig. 3) and the same amounts of ITSN1 were immunoisolated (Fig. 4). A stCCV contains 30% of wt, stable CCV ITSN1 and thus we expected to isolate less ITSN1 from stCCV, but stCCV eluates contained even slightly more ITSN1 than wt, stable CCV. Thus, ITSN1 immunoisolation efficiencies are CCV specific, for yet unkown reasons. The anti-ITSN1 antibody-AP2 competition for ITSN1 binding certainly contributes to this result^73^. However, the amounts of co-isolated synaptojanin1 are CCV specific and thus the protein ratios do demonstrate CCV specific protein:protein interaction preferences. A ko synapse canCCV contains 40% and a stCCV contains 55% of wt canCCV and stable CCV endophilin levels, but relatively more endophilin was co-isolated from both ko synapse CCV classes than from wt synapse CCV. Importantly, there is no difference in isolated endophilin levels between the wt and ko canCCV and the wt, stable CCV and the stCCV. This indicates a higher percentage of ITSN1-endophilin complexes in ko synapse CCV. This could be linked to the twofold stimulation of both CME pathways. Also these data speak against an endophilin function in the regulation of synaptojanin1 CCV levels.

A ko synapse canCCV appears to have more ITSN1-synaptojanin1 complexes than a wt synapse canCCV, although the ko canCCV contains 50% less synaptojanin1 than a wt canCCV. This supports a critical function of ITSN1 in regulating synaptojanin1 recruitment. Immunoisolation values indicate that stCCV have slightly fewer ITSN1-synaptojanin1 complexes than wt, stable CCV (Fig. 4). However, a stCCV contains only 10% of synaptojanin1 of a wt, stable CCV. Therefore, much less synaptojanin1 would be isolated, if the ITSN1-synaptojanin1 binding preference in stCCV would be identical with the preference in wt, stable CCV. Therefore, this experiment emphasises the regulation of synaptojanin1 levels by ITSN1 in CCV.

### AP2 co-adaptors in ITSN1 CCV incorporation

**Figure 5:**
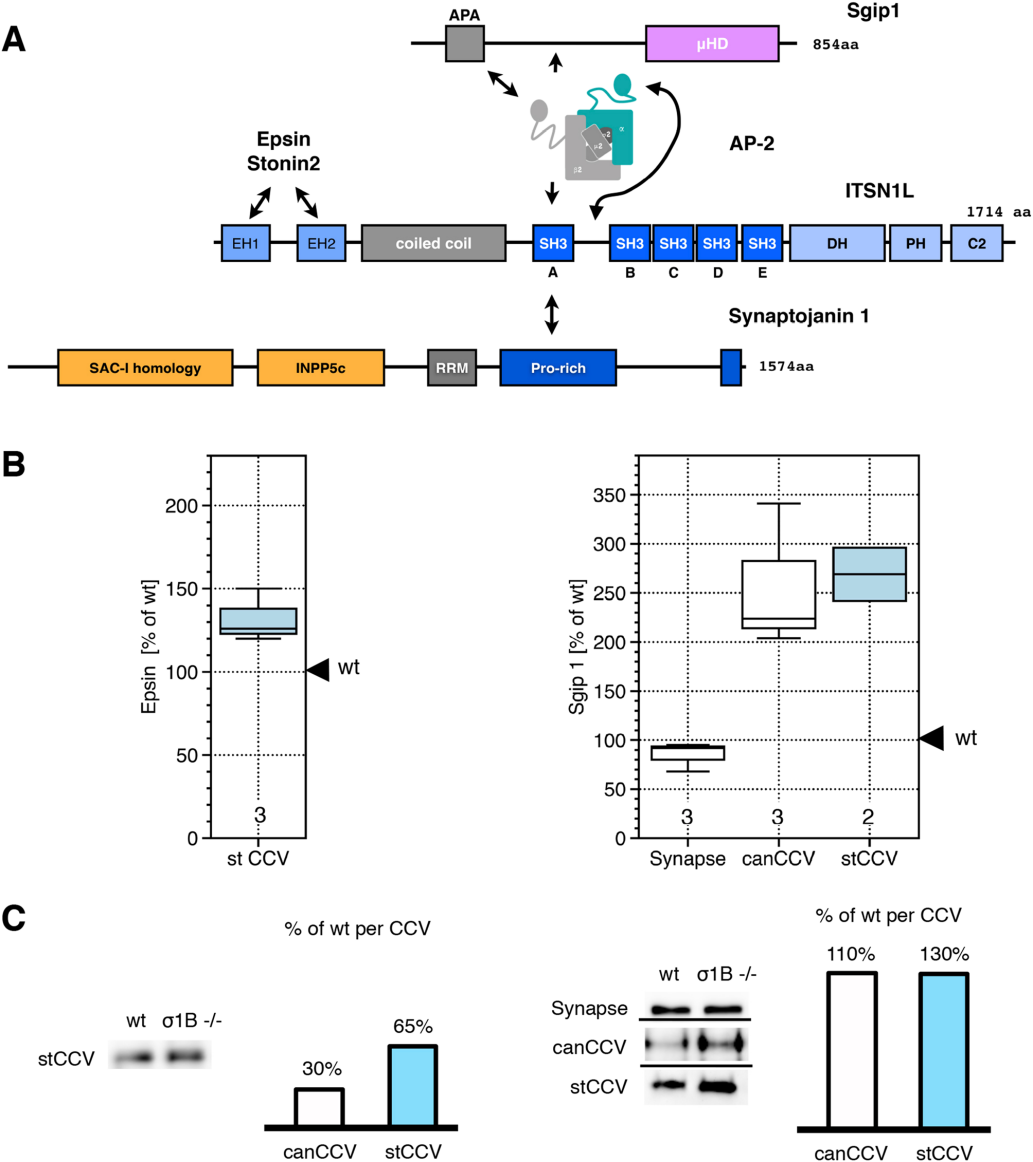
(**A**) Epsin, Sgip1 and AP2 binding of ITSN1 and competition with synaptojanin1. Epsin and stonin2 bind ITSN1 EH-domains. Sgip1 binds ITSN1-SH3A, as does synaptojanin1. AP2 α-adaptin ‘ear’ domain binds ITSN1 between SH3A-SH3B domains and the β2-adaptin ‘ear’ domain binds the APA domain of Sgip1. (**B**) For epsin only stCCV levels are shown, because synapse and canCCV data have been published previously (see text). Alterations in Sgip1 levels in brain subfractions of AP-1/σ1B ko mice. Changes are expressed relative to wt brain subfraction levels, defined as 100%. Numbers indicate the repeats of independent biological wt-ko pairs. (**C**) Representative western-blot experiments used for the box-blot diagrams (see “Materials and methods” section for details). Epsin, 85 kDa band; Sgip1, 95 kDa band. Bar schemes indicate changes in protein levels per CCV.

Why do stCCV have so much less ITSN1? ITSN1 is bound by proteins initiating CCV formation: AP2, Eps15, Eps15L1, epsin and Sgip1 (Figs. 1B, 5A). Tripartite Sgip1-AP2-Eps15 and -Eps15L1 complexes support initiation of AP2 CCV budding, because Sgip1 binds low curvature membranes with highest affinity^75–79^. Epsin interacts with AP2 and ITSN1^80–85^. Sgip1 binds, like synaptojanin1, to the ITSN1 SH3A domain. AP2 binds the ITSN1 SH3A-SH3B linker domain. All these proteins may compete for ITSN1 binding^73^. Eps15 and Eps15L1 bind the ITSN1 coiled-coil domain. Epsin and stonin2 bind the ITSN1 EH-domains^74^. Thus, protein:ITSN1 ratios of one of these proteins should determine the amount of ITSN1 in a CCV and thus we compared their levels between wt and ko CCV.

### ITSN1 EH-domain binding proteins do not regulate ITSN1 CCV levels

Stonin2 CCV levels were compared previously^1,2^. It is present at wt levels in stCCV and therefore is not limiting for ITSN1recruitment.

Epsin binds PI-4,5-P_2_ and ITSN1 (Fig. 5), AP2 and clathrin^80,81,83,86,87^. Epsin binds ubiquitinated cargo via its UIM domain (ubiquitin-interacting motif)^85,88^. Its ENTH domain dips in the cytoplasmic leaflet of the membrane, lowering the energy required for membrane bending^89^. CME is active in epsin ko MEF cells^90^. Thus, its prime function is to capture specific cargos^91^. We reasoned that its CCV levels might be even increased, because AP180, whose ANTH domain functions like the ENTH domain^89^, is reduced in ko canCCV to 60%, and in stCCV to 50% of wt amounts^1,2^. Increased epsin levels could compensate for lower AP180 levels. AP1/σ1B ko synapses have slightly more epsin (115%) compared to wt synapses and a canCCV from ko mice has 30% of epsin compared to wt canCCV^2^. A stCCV has 65% of the epsin level of a wt, stable CCV (Fig. 5). Changes in epsin and in ITSN1 CCV levels go in opposite directions and thus epsin does not regulate ITSN1 recruitment. These data also demonstrate that AP2 CCV pathways are not enhanced to endocytose more epsin cargo proteins.

### Sgip1 CCV levels are increased

Sgip1, a brain-specific homolog of the ubiquitous FcHO1/2 BAR-domain protein, binds low curvature membranes with highest affinity, influencing CCV budding kinetics^92,93^. AP1/σ1B ko mouse synapses contain essentially wt amounts of Sgip1 (Fig. 5B). An AP1/σ1B ko mouse canCCV contains wt Sgip1 levels. stCCV contain even more Sgip1, than wt, stable CCV (Fig. 5B,C). Sgip1 levels match the AP2 CCV levels, indicating identical budding kinetics for both CCV classes in ko synapses. Thus, Sgip1 does not determine CCV levels of ITSN1. Thus Eps15 and Eps15L1 might regulate ITSN1 recruitment and the CCV life cycle (Fig. 1).

### Eps15, not Eps15L1, CCV levels regulate ITSN1 recruitment

**Figure 6:**
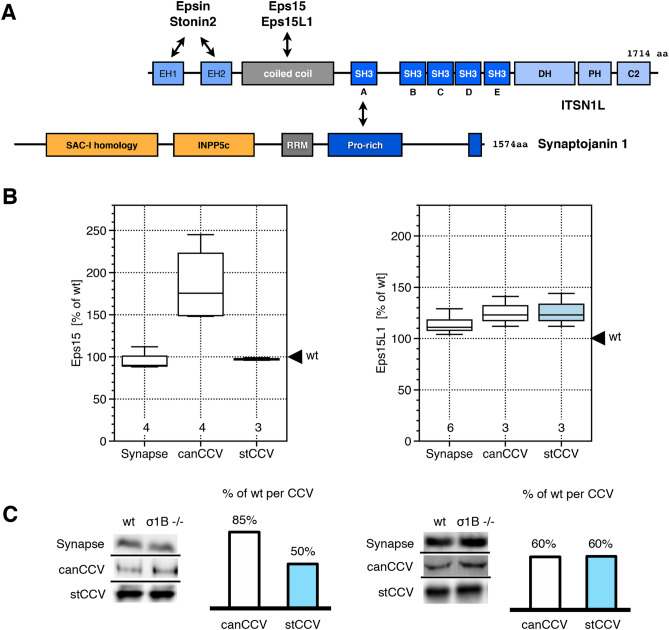
(**A**) ITSN1 domains bound by Eps15 and Eps15L1. (**B**) Alterations in Eps15 and Eps15L1 levels in brain synapses and synaptic CCV of AP-1/σ1B ko mice. Changes are expressed relative to wt brain subfraction levels, defined as 100%. Numbers indicate the repeats of independent biological wt-ko pairs. (**C**) Representative western-blot experiments used for the box-blot diagram (see “Materials and methods” section for details). Eps15, 140 kDa band, Eps15L1 (Eps15R), 74 kDa band. Bar schemes indicate changes in protein levels per CCV compared to wt set to 100%.

Due to the formation of Sgip1-AP2-Eps15 and -Eps15L1 complexes, Eps15 and/or Eps15L1 levels might be increased like the Sgip1 and AP2 levels. Eps15 and Eps15L1 bind the ITSN1 coiled-coil domain (Fig. 6)^35^. Eps15 ko mice are generally healthy and no strong neurological phenotype has been described, although it is required for SV recycling^94–96^. Eps15L1 ko mice die within two days after birth, showing severe neurological deficits. Thus, Eps15L1 appears to be able to substitute Eps15, but not vice versa^97^. Therefore, we expected CCV specific alterations in Eps15L1 rather than Eps15.

**Figure 7:**
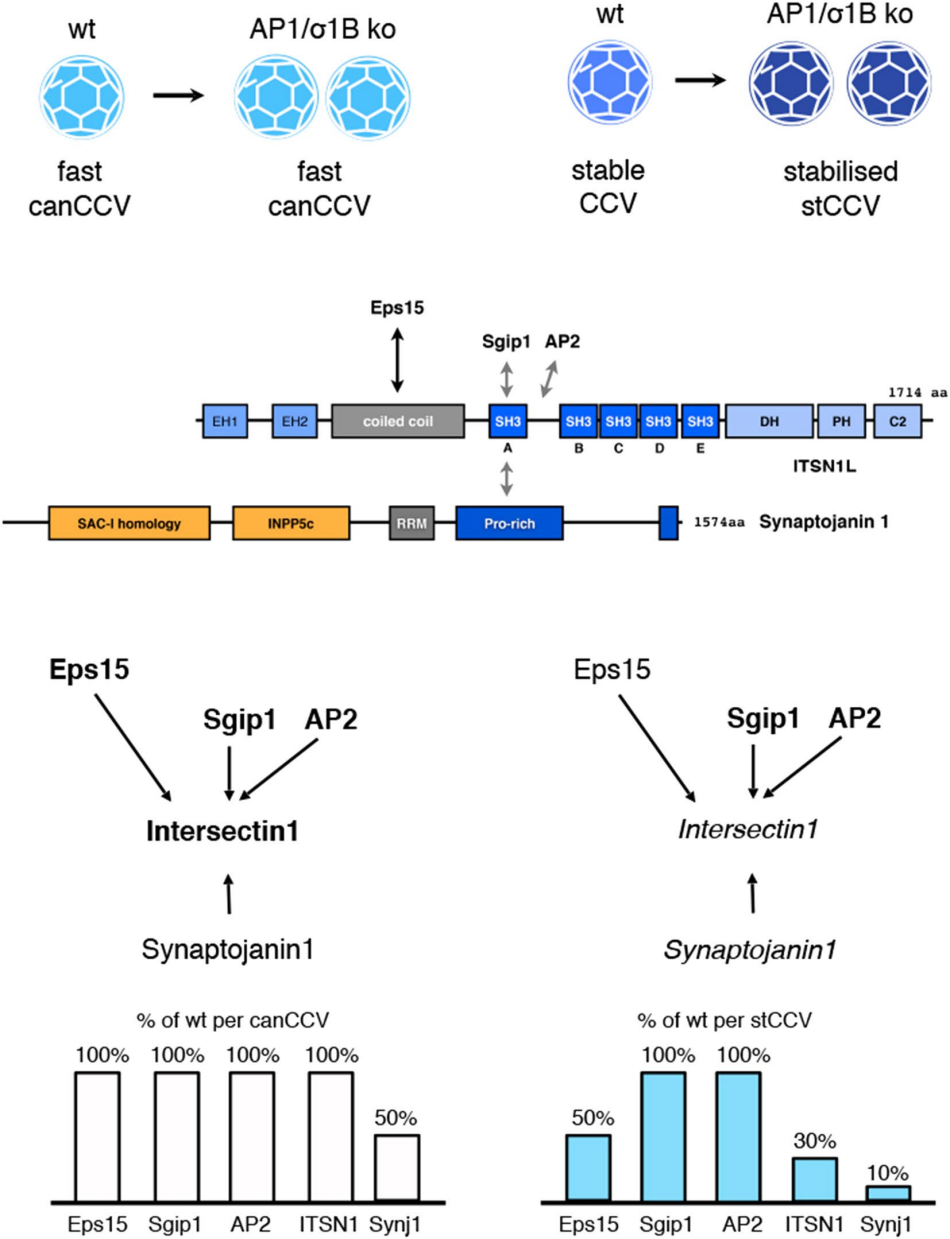
Model of protein–protein interactions determining the amount of synaptojanin1 stably incorporated into canCCV (left) and stCCV (right). Eps15 determines ITSN1 levels and the competition of Sgip1/AP2 and synaptojanin1 for ITSN1 determines synaptojanin1 levels. Interaction scheme: proteins in bold letters are present at 100% of wt levels, proteins in regular letters are reduced to 50% of wt levels, proteins in italics are reduced below 50% of wt levels. Please refer to the “Results” and “Discussion” sections for more details.

Eps15 levels are not altered in AP1/σ1B ko mouse synapses and also not in their canCCV (Fig. 6). A stCCV contains only 50% of the wt, stable CCV Eps15 level. Eps15L1 levels are slightly increased in ko mice synapses (Fig. 6). Both CCV classes of ko mouse synapses contain 65% of wt Eps15L1 levels. Although reduced, Eps15L1 incorporation into the CCV classes is not differentially regulated. Despite their high sequence homology, only Eps15 recruitment into the CCV classes is differently regulated. Both are modified by phosphorylation, ubiquitination and acetylation and differential modifications might be responsible for this isoform specific regulation^98,99^. Of all ITSN1 binding CCV proteins, only Eps15 levels are specifically reduced in stCCV and thus Eps15 levels areis limiting for ITSN1 recruitment in a CCV (Fig. 7).

## Discussion

We describe the analysis of synaptojanin1 recruitment in the AP2 CCV coat, a mechanism which regulates the AP2 CCV life cycle. We have a unique experimental approach. We are using a mouse ko model, in which the AP2 CCV life cycle is modified as a secondary, indirect phenotype of the ko of the tissue-specific subunit σ1B of the AP1 complex. The AP1/σ1B ko causes alterations in synaptic protein sorting, which stimulate AP2 CCV mediated endocytosis and in addition the formation of a population of longer lived AP2 CCV.

AP1/σ1B deficiency causes severe learning and memory deficits in mice and severe intellectual disability in humans^8^. Synapses of ko mice have impaired SV recycling, reduced SV numbers and large early endosomes, whose maturation into late, MVB endosomes is stimulated^1,2,7,8^. The ko synapses have a twofold increase in the number of synaptic AP2 CCV^2,100^. This increase is synapse specific and therefore a mechanism of synaptic plasticity. Synapses contain two classes of AP2 CCV, canonical, canCCV (~ 85%), a second class (~ 1 5%) with a more stable coat. Both classes are increased twofold in the ko mice. A second induced mechanism of AP2 CCV plasticity is the enhanced stabilisation of the class of stable CCV in ko synapses, named stCCV. stCCV will have a longer half life and thus these endocytic vesicles are unable to fuse with an acceptor membrane shortly after their formation. They have a specific role in AZ plasticity^1,2,7,8^. These alterations in the AP2 CCV pathways are expected to suppress the deficits in synaptic signalling and brain functions caused by impaired AP1-dependent protein sorting.

The alterations in the AP2 CCV life cycle are physiological modifications of AP2 CCV composition. This enables us to study the mechanisms determining the composition of a mature AP2 CCV, without mutating CCV coat proteins. Overexpression or ko of CCV proteins allow only to study their earliest functions in the pathway, because coat proteins interact with several coat proteins during CCV formation and thus, multiple interactions are disturbed.

AP2 CCV pathway specific regulation of synaptojanin1 recruitment in CCV is one of the three described molecular mechanisms responsible for the formation of the stCCV: reduced recruitment of the uncoating ATPase Hsc70, hyperactivation of AP2 via increased amounts of AAK1 kinase and μ2 phosphorylation and thirdly, reduced recruitment of the PI-4,5-P_2_ phosphatase synaptojanin1^1^. Synaptojanin1 dysfunctions induce several, severe neurological diseases, including Alzheimer’s and Parkinson’s diseases and Down syndrome^14,25,26,101,102^. This demonstrates the importance of the tight regulation of synaptojanin1 activities for brain function. Synaptojanin1 catalytic activity is stimulated by high membrane curvature^20^. Therefore, mechanisms regulating its recruitment into CCV are most important.

Synaptojanin1 interacts with 8 ubiquitously expressed CCV proteins, suggesting recruitment by several protein–protein interactions. Many of these proteins bind to each other forming a complex network (Figs. 1, 6). Of all the synaptojanin1 binding CCV proteins, endophilin has been considered to be most important for synaptojanin1 recruitment and CCV uncoating, because it binds shortly before membrane scission together with synaptojanin1^13,43,58^. It also appears reasonable, that an uncoating enzyme is recruited just before CCV formation is completed. However, endophilin CCV levels are not differentially altered between the CCV classes as are the synaptojanin1 levels (Fig. 1)^1^. Our data also speak against AP2 CCV class specific regulation of endophilin protein interactions by LRRK2 kinase, whose mutations are very often the cause for Parkinson’s disease^52–54,103,104^. However, our data do show that other endophilin CCV protein interactions do contribute to CCV life cycle regulation, because its binding to ITNS1 is enhanced in both ko CCV classes compared to wt CCV. This increased endophilin-ITSN1 interaction could be linked to the activation of both AP2 CCV pathways.

Pacsin1 is a synaptojanin1 binder, which connects CCV with the actin cytoskeleton^64,67,105^. Both canCCV contain the same amount of pacsin1,but stCCV contain only 30% of a wt, stable CCV However, this smaller pacsin1 pool has a higher phosphorylation and thus activation ratio, than pacsin1 of wt, stable CCV. The compensation of lower pacisn1 levels by an increased activation level is not in line with an impaired function and reduced synaptojanin1 recruitment into stCCV. We are not able to completely exclude a pacsin1 contribution to the regulation of synaptojanin1 recruitment. Unfortunately, the anti-pacsin1 antibodies do not allow efficient pacsin1 immunoprecipitation and the quantification of co-isolated proteins by semi-quantitative western-blot experiments. The same experiment we did to verify the function of ITSN1 (Fig. 4). The pacsin1 data demonstrate remarkable complex regulatory mechanisms controlling CCV maturation. This quality control, sensor kinase has to be identified. Currently, we are analysing the additional two mechanisms responsible for stCCV formation, reduced Hsc70 binding and increased AAK1 kinase binding. We hope to identify also this pacsin1 kinase in the course of these studies. Pacsin1 recruitment into stCCV is probably limited by an enriched coat protein, possibly the large scaffolding protein Git1.

The alterations in the CCV levels of the constitutively active synaptojanin1 binder ITSN1 do match those of synaptojanin1. We verified that ITSN1 is indeed the major CCV protein binding synaptojanin1 by immunoisolation of ITSN1 and determining the amounts of co isolated synaptojanin1. Importantly, the coat protein solutions used were from stripped CCV. Therefore, CCV proteins were present at the protein–protein ratios of a mature CCV and in their, possibly, post-translationally modified forms (see pacsin1 data). ITSN1 binds synaptojanin1 and endophilin independend of each other and it has been proposed, that ITSN1 coordinates the binding of both with each other and to the membrane^58^. Our data show that ITSN1 binds synaptojanin1 to regulate its CCV level and thus it takes part in the regulation of the CCV life cycle. The amount of synaptojanin1 recruited by ITSN1 can be lowered by ITSN1 interaction with AP2/Sgip1. Sgip1 binds the ITSN1 SH3A domain, as does synaptojanin1, and AP2 the SH3A-SH3B linker sequence. It has been proposed that they and synaptojanin1 compete for ITSN1 binding (Figs. 5, 6)^58^. Sgip1 and AP2 are present in excess over ITSN1 in stCCV and thus are most likely responsible for the further reduction in synaptojanin1 in stCCV. Synaptojanin1 binding by the other CCV coat proteins is compared to ITSN1, either of to low affinity to determine the amount of synaptojanin1 incorporated into CCV or the respective binding sites are sterically blocked or these proteins are modified and synaptojanin1 binding is inhibited. There interactions with synaptojanin1 could still have functions during CCV budding.

The next question to answer was, why do stCCV have so much less ITSN1 than wt, stable CCV? Of all possible ITSN1 recruiting CCV proteins, only the amount of Eps15 is differentially regulated between canCCV and stCCV. Eps15 and Eps15L1 bind the coiled-coil domain of ITSN1, located between EH and SH3 domains. This prevents ITSN1 homodimerisation and its inactivation^74,106^. It is remarkable that Eps15L1 is not involved in ISTN1 recruitment. This is surprising, because Eps15L1 ko mice show severe neurological defects, whereas Eps15 ko mice have comparably mild phenotypes. It seems that Eps15L1 is able to replace Eps15 in virtually any pathway^97^. Positions of functional domains are almost identical in Eps15 and Eps15L1 and thus there numerous phosphorylation and ubiquitination modifications should regulate isoform specific functions. The 50 amino acid long insertion in the Eps15L1 sequence is most frequently modified (summarised on PhosphoSitePlus). Analysis of coat proteome phosphorylation and dephosphorylation levels under different stimulation conditions of SV recycling, demonstrated functions in CCV and SV life cycle regulation^107^. Modifications might play a role in the regulation of CCV formation, but also in the regulation of the binding of specific cargo proteins^98,99^. Eps15 is also a cargo co-adapter CCV protein and therefore Eps15 specific cargo might determine the amount of Eps15 recruited into CCV and Eps15 ability to recruit ITSN1. Alternatively, cargo and other coat proteins might interfere with Eps15 recruitment into CCV and/or its ability to bind ITSN1. For example, the stonin2 mediated recruitment of the large scaffolding protein Git1 into the stCCV endocytic pathway could alter coat protein–protein interactions^1,3^. This has to be tested in the future.

Collectively, our data lead to the model (Fig. 7), that Eps15 controls the amount of ITSN1 stably incorporated into CCV and that ITSN1 determines the amount of synaptojanin1 stably incorporated into AP2 CCV. Finally, the Sgip1/AP2:ITSN1 ratios determine how much synaptotagmin1 can stably bind to the ITSN1 incorporated in a CCV.

We discovered the variability of AP2 CCV life cycles as a mechanism of synaptic plasticity. The proteins involved are ubiquitously expressed and thus comparable mechanisms may play important roles in other tissues as well. However, the pool sizes of stabilised and longer lived CCV may be even smaller and more difficult to identify due to lower pathway activities. Synaptic plasticity is related to mechanisms in cell plasticity during development, which mediate cell–cell contact and tissue formation. Therefore, differential AP2 CCV life times may also be important during development and in tumors. The model of AP CCV coat protein interactions deduced from the presented data describes how AP2 co-adaptor proteins and thus AP2 CCV cargo-proteins determine the life cycle of their CCV.

## Materials and methods

### Isolation of synaptic canCCV and stCCV

The AP1/σ1B ko mouse has been described^8,100,108^ and also the isolation of synaptosomes and synaptic CCV has been described, which followed established protocols^1,2^. Animals are kept at the central animal facility of the University Medical Center, University Göttingen according to international guidelines. Animals were killed with CO_2_ and cervical dislocation in accordance with the appropriate guidelines. Animal housing and the protocol for killing the animals were approved by the, Niedersächsisches Landesamt für Verbraucherschutz und Lebensmittelsicherheit’ (LAVES). Isolations from wt and AP1/σ1B−/− cortices were always performed in parallel and only data from wt and ko extracts prepared in parallel were compared with each other. Brains were isolated from 4 to 6 month old animals in late afternoon, snap frozen in liquid nitrogen and stored at − 80 °C. Isolation of stable CCV from wt and of stCCV from ko mice required 3 brains per genotype: sucrose density gradient fractions containing the purified synaptic CCV were pooled, and incubated with protein G Sepharose bead slurry (Protein G Sepharose 4 Fast Flow GE Healthcare) at 4 °C for 1 h. Beads were pelleted at 2000 rpm and the supernatant was incubated with 5 μg of anti-Hsc70 mouse monoclonal antibody (Synaptic Systems, Göttingen, Ger) over night at 4 °C. Protein G Sepharose beads were added at 4 °C for 4 h. The harvested beads were washed twice with CCV buffer and resuspended in 40 μL 3× SDS-PAGE loading buffer. The protein content of the beads and the wash fractions elution 1 and elution 2 were analysed by semi-quantitative western-blot analyses.

### CCV coat protein quantification

Brain extracts were prepared from wt and ko mice in parallel and comparisons of protein content were only made between extracts prepared in parallel, which were separated next to each other on the same SDS-PAGE and transferred onto a nitrocellulose membrane (GE Healthcare Protran, 0.45 μm). All wt/ko data pairs are from independent biological samples. Comparing the data from different animals and independent preparations requires a normalisation and thus wt values were defined as 100%. The protein load was varied between 10 and 80 μg per lane to determine the linear protein/chemiluminescence signal ratio. Several proteins of different molecular masses were detected on one western-blot nitrocellulose membrane. This served as internal control for protein isolation and detection by the ECL luminescence detection kits PICO, NANO, FEMTO (Pierce-ThermoScientific, Karlsruhe, Ger), recorded with a Fuji LAS 1000 (Fujifilm Corp., Düsseldorf, Ger) camera system. Protein determination: Bradford-assay (BioRad, Munich, Ger) (Supplementary Material).

#### Coat protein: protein interactions

canCCV, wt stable CCV and stCCV were purified as described above. CCV coat disassembly of canCCV and of anti-Hsc70 antibody beads bound wt stable CCV and stCCV was induced by a pH shift from 6.4 to 8.0 in CCV buffer and incubation over 15 min at RT. Vesicles, beads and solubilised proteins were separated by centrifugation at 100.000×*g* for 15 min at 4 °C (Beckman table-top ultracentrifuge). Supernatants containing solubilised CCV proteins were transferred into new tubes and the pH was adjusted back to pH 6.4 to reestablish the conditions stabilising CCV protein:protein interactions. 5 μg of the ITSN1 antibody was added to 1800 μL supernatant and incubated overnight at 4 °C. Protein-antibody complexes were isolated with Protein G sepharose-beads (GE Healthcare Life Sciences). Protein loaded beads were washed three times with pH 6.4 CCV buffer. Proteins were eluted by adding non-reducing, dye containing, 3× SDS-PAGE protein loading buffer. Elution fractions from 3 wt and 3 ko CCV-coIP isolations were pooled and loaded next to each other on SDS-PAGE gels and semi-quantitative western-blot analyses were performed as described above.

#### Statistics

All experiments were performed according to ARRIVE guidelines. The experiments were repeated at least three times with independent biological samples as described above. Wild-type and ko brain extracts were handled in parallel and values from wild-type samples were defined as 100% and the values of the ko samples processed in parallel are expressed relative to these 100%. Only data sets in which all ko values are either above or below the respective wild-type values are considered to be significant. Statistics of the quantifications are presented as bar-plot diagrams, generated with DataGraph (Visual Data Tools, USA).

#### Antibodies

Antibodies: anti-α-adaptin (AP2) (1:1000), anti-Eps15 (1:1000) and anti-ITSN1 (1:1000) were from BD Biosciences; anti-dynamin (1:1000), anti-Hsc70 (1:1000), anti-synaptojanin1 (1:300), its splice-variant (1:500), anti-endophilin A1 (1:1000) and anti-pacsin1 (1:1000) were from Synaptic Systems; anti-pacsin1 Ser346-Pi (1:1000) Merck Millipore; anti-LRRK2 (1:1000) Novus Biologicals, anti-ArhGEF7 (1:1000) GeneTex; anti-LRRK2 Ser935-Pi (1:1000) and anti-epsin1 (1:500) were from Abcam; anti-Sgip1 (1:500) Acris; anti-Eps15L1 (Eps15R) (1:500) Biorbyt; HRP-conjugated antibodies (1:10000): Dianova (Hamburg, Ger), anti-mouse (product # 111-035-144), anti-rabbit (product # 115-035-062); anti-goat (1:5000) (product # 305-035-045).

## Supplementary Information

Supplementary Information.
