# Supplementary material for: Synaptic AP2 CCV life cycle regulation by the Eps15, ITSN1, Sgip1/AP2, synaptojanin1 interactome
Source: Sci Rep. 2021 Apr 13;11:8007. doi: 10.1038/s41598-021-87591-3 (PMC8044098; doi:10.1038/s41598-021-87591-3)

Supplementary Information

Synaptic AP2 CCV life cycle regulation by the Eps15, ITSN1, Sgip1/AP2, synaptojanin 1 interactome

by

Mishra, R.[1], Sengül, G.F., Candiello, E.[2] and Schu, P.*

Georg-August University Göttingen, University Medical Center, Department of Cellular Biochemistry, Humboldtallee 23, 37073 Göttingen, Germany

Images show the western-blot membranes used for signal quantifications and from which we selected the representative western-blot bands to be shown in the figures of the main manuscript. Membranes were cut to allow the detection of several proteins from the same experiment. This served also as internal control for the wt - ko protein samples.

Please refer to the results and materials & methods sections of the main manuscript for informations concerning the experimental design and experimental details.

LRRK2

synapse (RM)

wt ko wt ko wt ko

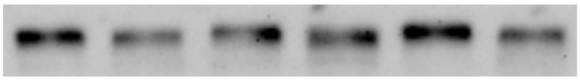

canCCV (GFS)

wt ko wt ko wt ko

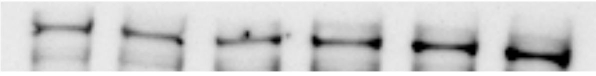

stCCV1 (RM)

Elu1 Elu2 beads

wt ko wt ko wt ko

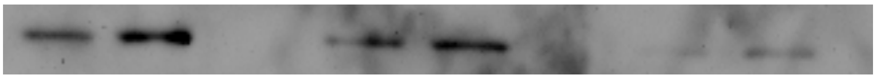

stCCV2 (RM)

Elu1 Elu2 beads

wt ko wt ko wt ko

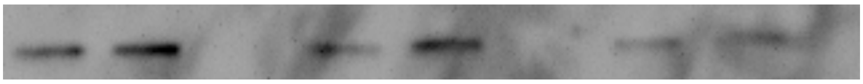

stCCV3 (RM)

Elu1 Elu2 beads
wt ko wt ko wt ko

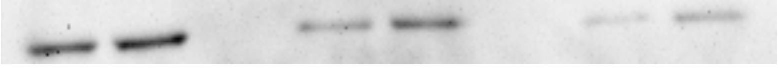

stCCV4 (RM)

Elu1 Elu2 beads
wt ko wt ko wt ko

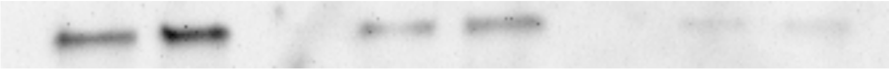

ArhGEF7

synapse (GFS)

wt ko wt ko wt ko wt ko

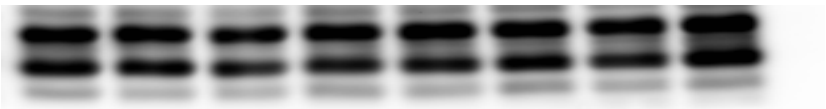

canCCV (GFS)

wt ko wt ko wt ko

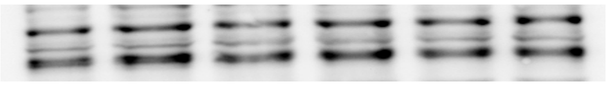

stCCV1 (GFS)

Elu1 Elu2 beads
wt ko wt ko wt ko

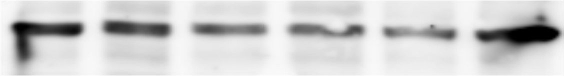

stCCV2 (GFS)

Elu1 Elu2 beads
wt ko wt ko wt ko

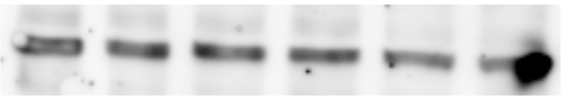

stCCV3 (GFS)

Elu1 Elu2 beads
wt ko wt ko wt ko

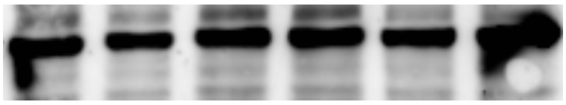

phospho-LRRK2

synapse (GFS)

wt ko wt ko wt ko

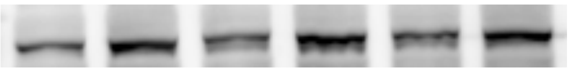

canCCV (GFS)

wt ko wt ko wt ko

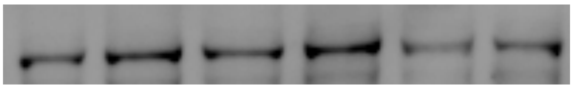

stCCV1 (GFS)

Elu1 Elu2 beads
wt ko wt ko wt ko

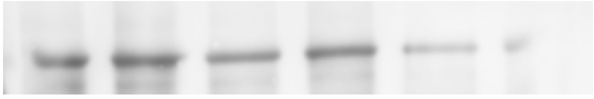

stCCV2 (GFS)

Elu1 Elu2 beads
wt ko wt ko wt ko

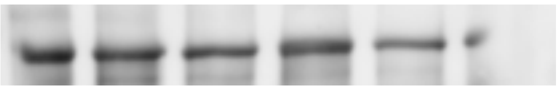

stCCV3 (GFS)

Elu1 Elu2 beads
wt ko wt ko wt ko

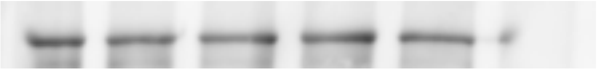

PACSIN1

canCCV (GFS)

wt ko wt ko

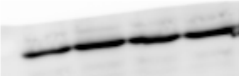

canCCV (EC)

wt wt wt wt ko ko ko ko

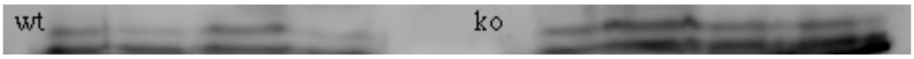

stCCV123 (EC)

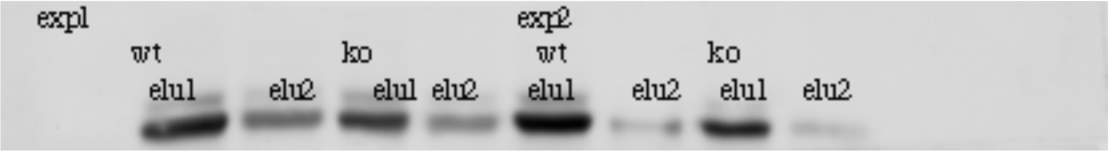

phospho-PACSIN1

canCCV (EC)

wt1 wt2 wt3 ko3 ko2 ko1

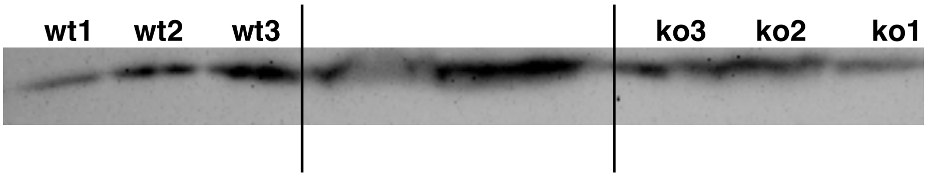

canCCV (GFS)

wt ko wt ko wt ko

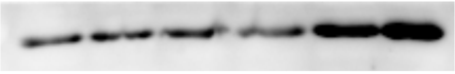

stCCV1 (EC)

wt ko

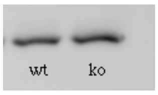

INTERSECTIN1

synapse (RM)

wt ko wt ko wt ko wt ko

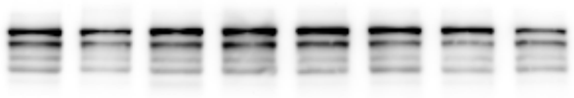

canCCV (RM)

wt ko wt ko wt ko

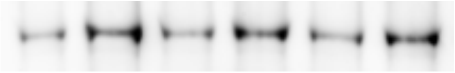

stCCV1 (RM)

Elu1 Elu2 beads
wt ko wt ko wt ko

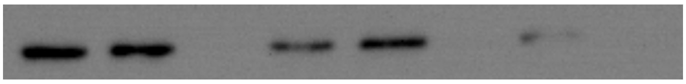

stCCV2 (RM)

Elu1 Elu2 beads
wt ko wt ko wt ko

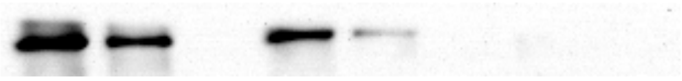

stCCV3 (RM)

Elu1 Elu2 beads
wt ko wt ko wt ko

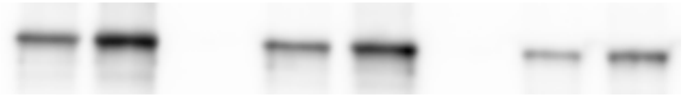

co-IP ITSN1

We could load only a small fraction (5% of total proteins) of the solubilised CCV coat proteins as the input-control for wt and ko CCV isolations. Due to low amount we had to collect signals for these bands over a longer period of time compared to the elution fraction. Thus signals from each western-blot membrane were collected for a short period (elutions) and for a longer period of time (input).

IP-ITSN1

canCCV1, ~5% input (GFS)

wt ko

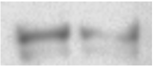

canCCV1, ELU (GFS)

wt ko

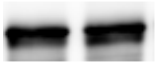

canCCV2, ELU (GFS)

wt ko

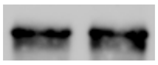

canCCV3, ~5% input (GFS)

wt ko

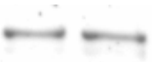

canCCV3, ELU (GFS)
wt ko

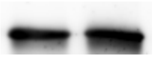

IP ITSN1

stCCV1, ~5% input (GFS)

wt ko

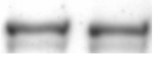

stCCV1, ELU (GFS)

wt ko

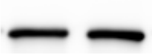

stCCV2, ~5% input (GFS)

wt ko

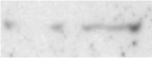

stCCV2, ELU (GFS)

wt ko

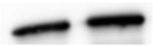

stCCV3, ELU (GFS)

wt ko

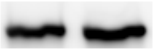

co-IP ITSN1

Synaptojanin1

canCCV1, ELU (GFS)

wt ko

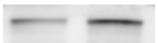

canCCV2, ELU (GFS)

wt ko

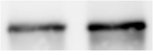

canCCV3, ELU (GFS)

wt ko

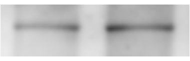

Synaptojanin1

stCCV1, ELU (GFS)

wt ko

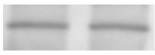

stCCV2, ELU (GFS)

wt ko

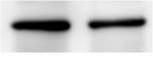

stCCV3, ELU (GFS)

wt ko

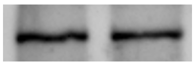

co-IP ITSN1

Endophilin A1

canCCV1, ELU (GFS)

wt ko

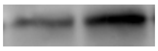

canCCV2, ELU (GFS)

wt ko

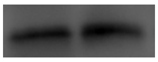

canCCV3, ELU (GFS)

wt ko

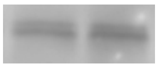

Endophilin A1

stCCV1, ELU (GFS)

wt ko

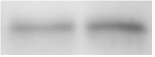

stCCV2, ELU (GFS)

wt ko

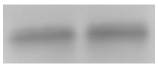

stCCV3, ELU (GFS)

wt ko

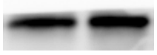

co-IP ITSN1

Dynamin123 (neg. control for co-IP ITSN1)

canCCV1, ~5% input (GFS)
wt ko

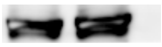

canCCV1, ELU (GFS)
wt ko

canCCV2, ~5% input (GFS)
wt ko

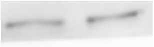

canCCV2, ELU (GFS)
wt ko

canCCV3, ~5% input (GFS)
wt ko

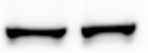

canCCV3, ELU (GFS)
wt ko

Dynamin123 (neg. control)

stCCV1, ~5% input (GFS)
wt ko

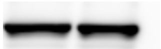

stCCV1, ELU (GFS)
wt ko

stCCV2, ~5% input (GFS)
wt ko

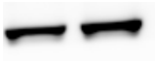

stCCV2, ELU (GFS)
wt ko

Epsin

stCCV1 (RM)

Elu1 Elu2 beads
wt ko wt ko wt ko

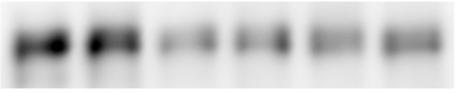

stCCV2 (RM)

Elu1 Elu2 beads
wt ko wt ko wt ko

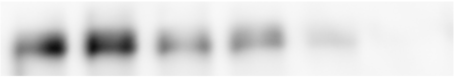

stCCV3 (RM)

Elu1 Elu2 beads
wt ko wt ko wt ko

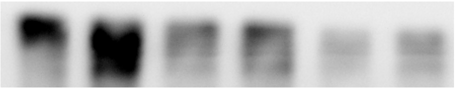

stCCV4 (RM)

Elu1 Elu2 beads
wt ko wt ko wt ko

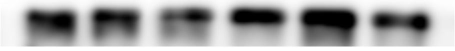

SGIP1

synapse (RM)

wt ko Ø Ø wt ko wt ko

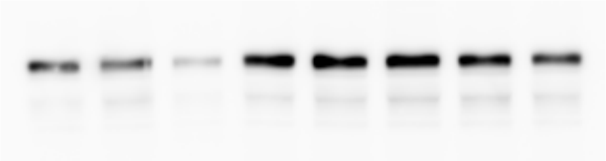

canCCV (RM)

wt ko wt ko wt ko

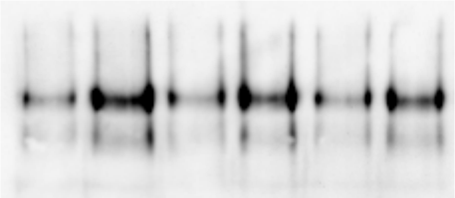

stCCV1 (RM)

Elu1 Elu2 beads
wt ko wt ko wt ko

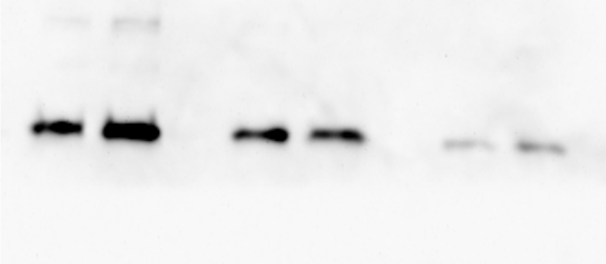

stCCV2 (RM)

Elu1 Elu2 beads
wt ko wt ko wt ko

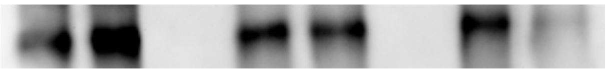

stCCV3 (RM)

Elu1 Elu2 beads
wt ko wt ko wt ko

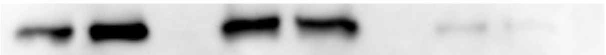

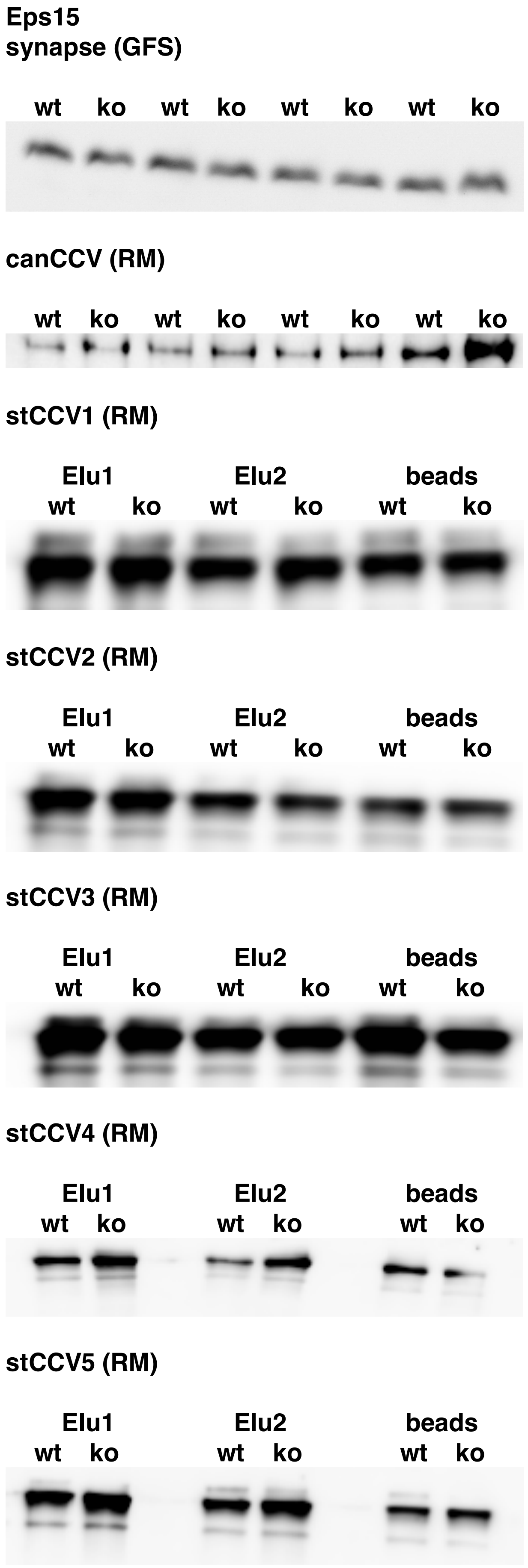
Eps15
synapse (GFS)
wt ko wt ko wt ko wt ko
canCCV (RM)
wt ko wt ko wt ko wt ko
stCCV1 (RM)
Elu1 Elu2 beads
wt ko wt ko wt ko
stCCV2 (RM)
Elu1 Elu2 beads
wt ko wt ko wt ko
stCCV3 (RM)
Elu1 Elu2 beads
wt ko wt ko wt ko
stCCV4 (RM)
Elu1 Elu2 beads
wt ko wt ko wt ko
stCCV5 (RM)
Elu1 Elu2 beads
wt ko wt ko wt ko

Eps15L1

synapse (GFS)

wt ko wt ko wt ko wt ko

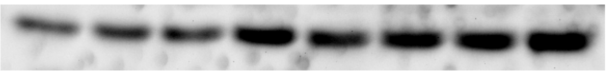

synapse (RM)

wt ko wt ko wt ko wt ko

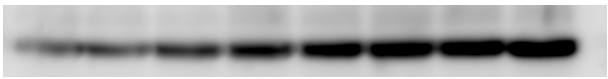

canCCV (GFS)

wt ko wt ko wt ko

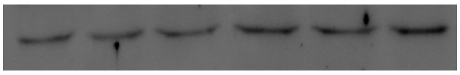

stCCV1 (RM)

Elu1 Elu2 beads
wt ko wt ko wt ko

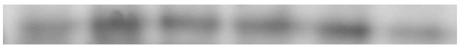

stCCV2 (RM)

Elu1 Elu2 beads
wt ko wt ko wt ko

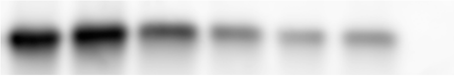

stCCV3 (RM)

Elu1 Elu2 beads
wt ko wt ko wt ko

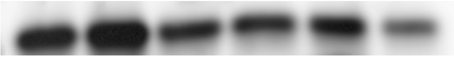

Supplement: Supplementary file 1 — Supplementary Information. [file 41598_2021_87591_MOESM1_ESM.pdf]
